# Approaches to identifying drug resistance mechanisms to clinically relevant treatments in childhood rhabdomyosarcoma

**DOI:** 10.20517/cdr.2021.112

**Published:** 2022-01-04

**Authors:** Samson Ghilu, Christopher L. Morton, Angelina V. Vaseva, Siyuan Zheng, Raushan T. Kurmasheva, Peter J. Houghton

**Affiliations:** 1 Department of Molecular Medicine, Greehey Children’s Cancer Research Institute, UT Health, San Antonio, TX 78229, USA.; 2 Department of Surgery, St. Jude Children’s Research Hospital, Memphis, TN 38105, USA.; 3 Department of Epidemiology and Biostatistics, Greehey Children’s Cancer Research Institute, UT Health, San Antonio, TX 78229, USA.

**Keywords:** Rhabdomyosarcoma, patient-derived xenografts, combination therapy, intrinsic drug resistance, acquired drug resistance

## Abstract

**Aim:**

Despite aggressive multiagent protocols, patients with metastatic rhabdomyosarcoma (RMS) have poor prognosis. In a recent high-risk trial (ARST0431), 25% of patients failed within the first year, while on therapy and 80% had tumor progression within 24 months. However, the mechanisms for tumor resistance are essentially unknown. Here we explore the use of preclinical models to develop resistance to complex chemotherapy regimens used in ARST0431.

**Methods:**

A Single Mouse Testing (SMT) protocol was used to evaluate the sensitivity of 34 RMS xenograft models to one cycle of vincristine, actinomycin D, cyclophosphamide (VAC) treatment. Tumor response was determined by caliper measurement, and tumor regression and event-free survival (EFS) were used as endpoints for evaluation. Treated tumors at regrowth were transplanted into recipient mice, and the treatment was repeated until tumors progressed during the treatment period (i.e., became resistant). At transplant, tumor tissue was stored for biochemical and omics analysis.

**Results:**

The sensitivity to VAC of 34 RMS models was determined. EFS varied from 3 weeks to > 20 weeks. Tumor models were classified as having intrinsic resistance, intermediate sensitivity, or high sensitivity to VAC therapy. Resistance to VAC was developed in multiple models after 2–5 cycles of therapy; however, there were examples where sensitivity remained unchanged after 3 cycles of treatment.

**Conclusion:**

The SMT approach allows for *in vivo* assessment of drug sensitivity and development of drug resistance in a large number of RMS models. As such, it provides a platform for assessing *in vivo* drug resistance mechanisms at a “population” level, simulating conditions *in vivo* that lead to clinical resistance. These VAC-resistant models represent “high-risk” tumors that mimic a preclinical phase 2 population and will be valuable for identifying novel agents active against VAC-resistant disease.

## INTRODUCTION

Advanced or metastatic rhabdomyosarcoma (RMS) has a poor prognosis. Patients with stage 4 disease, with the exception of those with embryonal RMS that are less than ten years of age, have a long-term event-free survival (EFS) of less than 20%^[1–3]^. Current therapies include all the known active agents plus radiation and surgery, but neither intensification of chemotherapy by increasing the dose of cyclophosphamide and adding active agents to standard VAC therapy (Vincristine/Actinomycin-D/Cyclophosphamide), nor use of high-dose chemotherapy with stem-cell rescue has improved outcome over the last 30 years^[4]^. The failure of current therapeutic approaches to effectively treat advanced or metastatic RMS despite numerous cooperative group trials, is a consequence of intrinsic or acquired resistance to the limited number of drugs that compose the armamentarium for treating this disease. Even with the use of intensive multimodality treatment, only 20% of patients will be long-term survivors. The failure of current therapies is further emphasized by the rapid progression rate of patients enrolled in a recent high-risk rhabdomyosarcoma study (ARST0431). In this trial, 25% of patients diagnosed with metastatic disease, either failed during the 52 weeks of treatment or had an event within two years (80%)^[5]^. The causes of tumor resistance are poorly understood. One possible cause for resistance is tumor clonal evolution, where novel mutations acquired during therapy confer resistance^[6]^; however, such studies usually rely on small cohorts as relatively few tumors are re-biopsied at relapse.

Preclinical studies aimed at understanding resistance tend to focus on individual drugs, rather than resistance to poly-chemotherapy^[7–13]^. Further, often drug exposures used *in vitro* far exceed those achieved in patients, and escalating drug concentrations over time do not simulate how clinical resistance is acquired^[14]^. We have previously used pediatric tumor xenografts to select for acquired resistance in mice^[7,8]^. However, while this approach is perhaps more appropriate than *in vitro* selection, conventional testing is resource-intensive, and allows for only a few models to be explored^[15]^. More recently, we have adopted Single Mouse Testing (SMT) to evaluate drugs in a large number of xenograft models^[16,17]^, an approach that significantly increases the inclusion of genetic diversity for a given cancer type^[18]^. The SMT design is validated by a retrospective analysis of > 2100 tumor-drug studies undertaken by the Pediatric Preclinical Testing Program, where the response of a tumor in one mouse, selected at random from the group, was compared to the median group response. This analysis showed that the SMT accurately predicted responses in 78% of studies. Allowing for a deviation of ± one response classification [e.g., stable disease (SD) *vs*. partial response (PR)], the concordance was 95%. Further, the SMT analysis was accurate in identifying the antitumor activity of 66 of 67 drugs in terms of the objective response rate determined for each drug over a range of tumor models^[18]^. Prospective studies with up to 90 ALL models and up to 50 solid tumor models show that SMT has similar concordance with conventional testing^[16]^. Importantly, using SMT, one can potentially incorporate up to 20-fold the number of models for evaluation of an agent, encompassing many diseases, or encompassing the genetic diversity of a given disease. Here we have applied SMT to interrogate intrinsic resistance and to use this approach to select for acquired resistance *in vivo* to VAC therapy in 34 RMS models. Our data show that RMS models have a range of sensitivities to VAC, ranging from tumor progression through initial treatment to maintained complete response at week 20 after a single cycle of VAC treatment. Further, using the SMT design, acquired resistance can be developed over a period of 3 to 5 cycles of therapy.

## METHODS

C.B.17SC *scid*^*−/−*^ (C.B-*Igh*-1^b^/IcrTac-*Prkdc*^*scid*^) female mice (Envigo, Indianapolis, IN) were maintained under barrier conditions, and experiments were conducted using protocols and conditions approved by the Institutional Animal Care and Use Committee at UTHSA as previously described^[15]^. Mice were selected for VAC treatment when tumors were 200–300 mm^3^. Regrowth of tumors was determined following tumor regression. Endpoints were EFS, defined as tumor growing to 400% of its volume at the initiation of treatment, and percent tumor volume regression. Complete regression (CR) was defined as tumor volume < 40 mm^3^ (the level of detection). Limited demographic data for RMS models used in the studies are presented in Table 1. Patient- and cell-derived xenograft models (PDX and CDX) have been described previously^[15,17]^. Genomic data for some of the models is under https://ocg.cancer.gov/programs/target/ Pan-cancer Model Systems, and in Rokita *et al*.^[19]^. SJRHB011_X, SJRHB013_X, SJRHB010927_X1, SJRHB000026_X1, and SJRHB013758_X1 were obtained from The Childhood Solid Tumor Network^[20]^. SMS-CTR cells were obtained from Dr. Corine Linardic, Duke University, JR1 (UK), and CCA cells were obtained from Dr.Marielle Yohe, NCI. All tumors were authenticated by short tandem repeat analysis against reference profiles.

### Simulating VAC therapy in mice

All drugs were administered IP. Vincristine was administered at 0.5 mg/kg days 1,8,15, actinomycin D (0.20 mg/kg) and cyclophosphamide (120 mg/kg), both on day 1, doses considered to give clinically relevant drug exposures^[21–23]^. All drugs were formulated in 0.9% w/v saline. This regimen, which simulates VAC modules used in ARST0431, was tolerated with ~10% body weight loss, and mortality < 2%.

### Development of acquired resistance in mice

**Figure 1.**
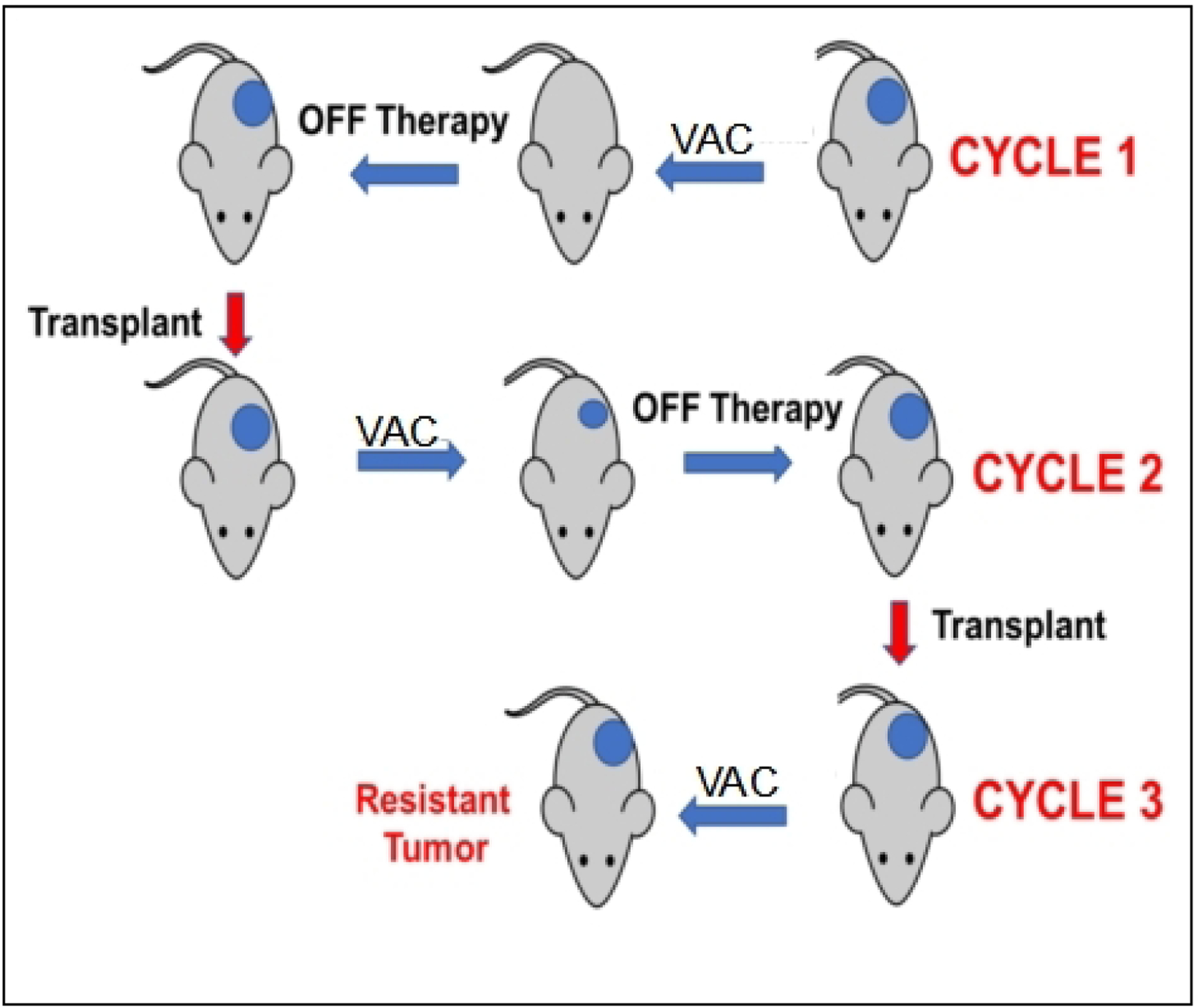
Schema for developing resistance to VAC therapy in mice using the single mouse study design. Mice received a single cycle of VAC (vincristine, actinomycin D, cyclophosphamide). Tumor response was determined, and tumors were transplanted in recipient mice when they achieved 400% of their volume on the first day of treatment.

The schema for selecting for acquired resistance is presented in Figure 1. Briefly, one mouse per tumor line received a single cycle of VAC treatment. Tumor diameters were measured weekly, and treated tumors were re-transplanted into new recipient mice when they “evented” (achieved 400% of their volume on day 1 of treatment). Treated tumors were considered resistant to VAC therapy if they progressed through treatment and had increased > 25% in volume by day 21. Tumor tissue was cryopreserved and snap frozen after each cycle of VAC therapy.

## RESULTS

### Intrinsic sensitivity to VAC treatment

**Figure 2.**
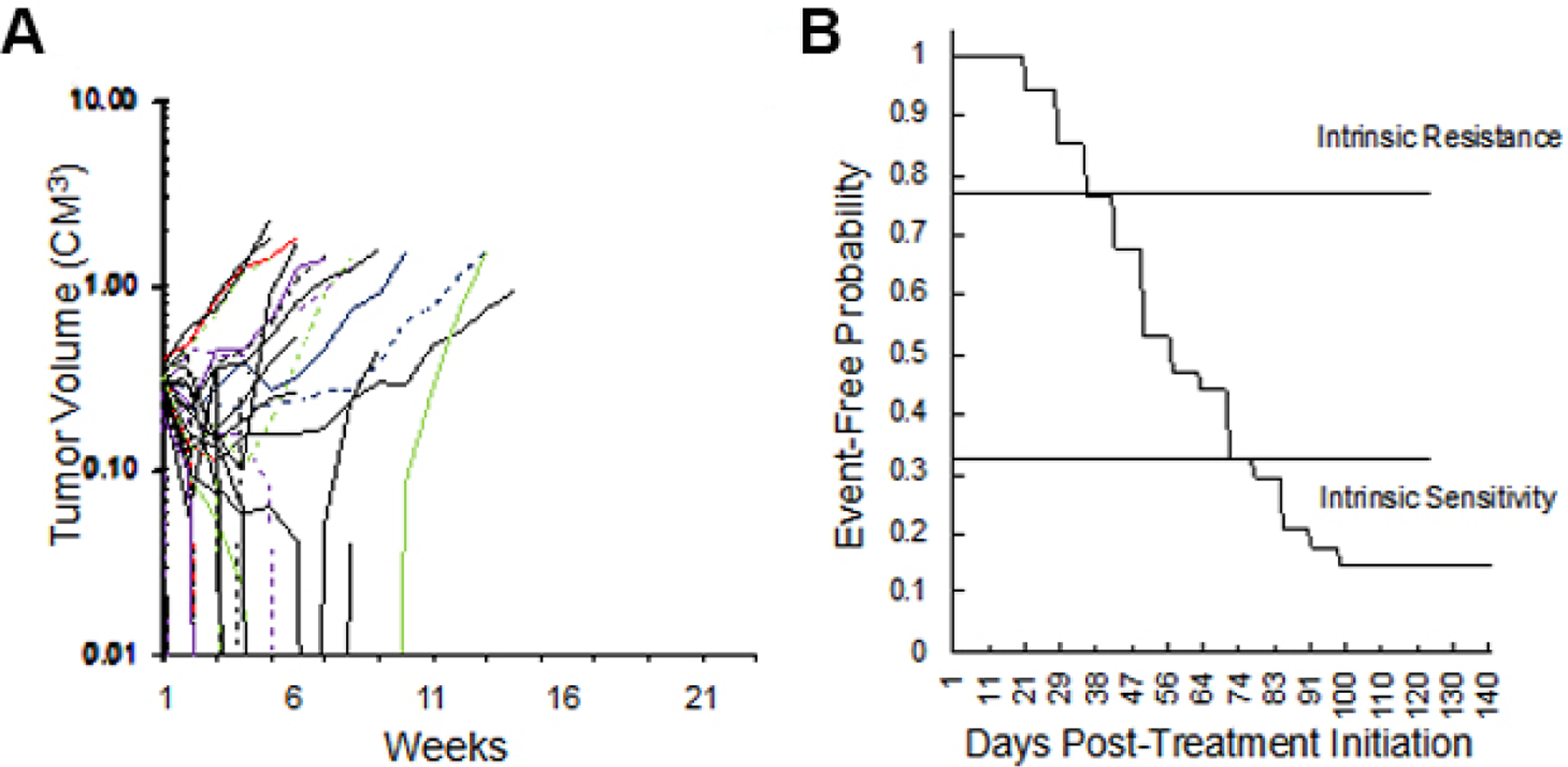
(A) Initial volume responses of 34 RMS models to cycle 1 of vincristine + actinomycin D + cyclophosphamide (VAC) using the SMT experimental design (ongoing expt.). (B) Kaplan-Meier plot for EFS of the same models. RMS: Rhabdomyosarcoma; VAC: vincristine/actinomycin-D/cyclophosphamide; EFS: event-free survival; SMT: single mouse testing.

We initially explored the sensitivity of 34 RMS xenograft models to the “gold standard” therapy for RMS, namely VAC, Figure 2. The models encompass both fusion-positive (alveolar), and fusion-negative (embryonal, with and without RAS mutations). As shown in Figure 2A, 18 of 34 xenograft models (53%) regressed > 50% on Cycle 1 of VAC therapy, the other models showing SD (*n* = 3) or progressive disease (PD) (*n* = 13). This experiment is ongoing, but clearly shows that SMT can be valuable for assessing drug sensitivity in a large cohort of models. Thirteen models were intrinsically resistant to VAC treatment with EFS of < 40 days, whereas others (*n* = 10) are highly sensitive (EFS > 70 days), with four models remaining in CR at 120 days, as shown in the Kaplan-Meier plot [Figure 2B]. We defined PD as tumors that increased in volume by 25% at day 21 (6 days after the last dose of vincristine). Thirteen of 34 RMS models (38%) exhibited PD to cycle 1 of VAC. An EFS of 49 days or less was also observed for 13 models, Table 1. Of these 13 intrinsically resistant models, 10 tumors were established from patients following treatment. The exceptions were Rh71, Rh85, and NCH-ERMS1, which were established as PDX models from patients prior to any treatment. Other models with short EFS (CCA, Rh36, IRS-68) each regressed > 50% (i.e., PR), but rapidly regrew. Of the models having EFS of 70 days or greater (*n* = 10; 29%), 3 models had CR (Rh88, SMS-CTR, SJRHB013x, and SJRHB013758_X1), 2 had PR (SJRHB010927, Rh83), and 1 had prolonged SD (Rh84). Three models, established from tumor at diagnosis, were highly sensitive to VAC treatment with no evidence of tumor regrowth at day 120 after the start of treatment (IRS-56, Rh12, Rh28). The other 11 models had intermediate sensitivity to VAC (EFS > 40 but < 70 days).

### Development of acquired resistance

**Figure 3.**
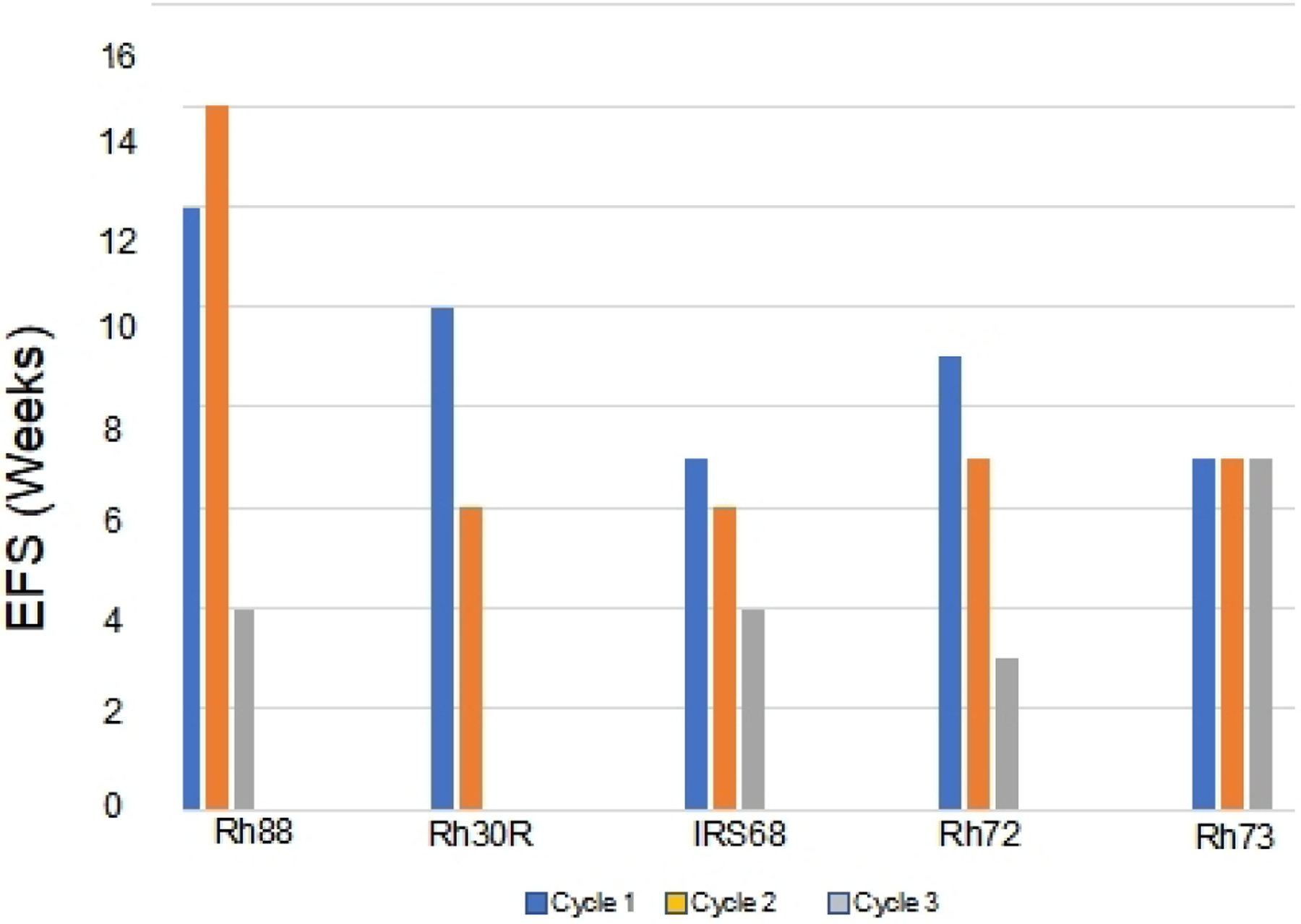
Development of resistance to VAC treatment (decreases in EFS) in models that were intrinsically sensitive to treatment (> PR; Rh88, Rh30R, IRS-68) or models where tumors progressed through cycle 1 of VAC treatment (Rh72, Rh73). VAC: Vincristine/actinomycin-D/cyclophosphamide; EFS: event-free survival; PR: partial response.

The approach to selecting for acquired resistance [Figure 1], was chosen as there is an increased incidence of scid lymphoma as mice age, and also potentially altered immunity that could alter the response to chemotherapy. Consequently, we re-transplanted tumors at 400% of their volume at day 1 of treatment into new 6 to 8-week-old female C.B.17SC *scid*^*−/−*^ mice, and repeated this procedure until tumors became resistant. For all studies, tumors were allowed to grow to 200–300 mm^3^ before starting treatment. Initially, we transplanted cycle 1 tumor into two mice, in case there was toxicity, and we would lose the model if there was a death (drug related or unrelated) on cycle 2 of treatment. However, there was no mortality, so subsequently, a single mouse design was used for all subsequent cycles of VAC treatment. Where two mice were used (*n* = 16), the responses between tumors were very similar, Supplemental Figure 1. The “Swimmer plot” in Figure 3 shows examples of models exhibiting Complete Regression (Rh88) or Partial Regression (IRS-6) to VAC treatment on cycle 1 with rapid emergence of resistance, or tumor models that initially had progressive disease (i.e., intrinsically resistant), but where EFS shortened with further cycles of VAC treatment (Rh72), or a model where EFS remained constant during 3 cycles of VAC treatment (Rh73).

## DISCUSSION

The outcome for patients with high-risk RMS has not improved significantly for several decades. In part, this is because new effective agents have not been identified, and agents such as irinotecan, while active against RMS^[24,25]^, do not increase overall survival when added to VAC therapy^[5,26]^. Of the chemotherapeutic agents used in high-risk protocols such as ARST0431, all but one (vincristine), induces DNA damage. However, the mechanism(s) that confer resistance to multi-chemotherapy protocols, even protocols that combine mostly DNA damaging agents, remains unknown. A proportion of patients failing on therapies including cyclophosphamide and vincristine^[27]^, however, may respond to vinorelbine/cyclophosphamide^[28]^.

Approaches to determine mechanisms of resistance, both *in vitro* and *in vivo*, have limitations. As mentioned previously, *in vitro* studies may not accurately replicate clinical drug exposures, and continuous exposure to increasing drug levels may result in multidrug resistance mechanisms, such as overexpression of ABC transporters, that may not reflect clinical reality^[29]^. In vivo studies in mice, using human tumor xenografts, or syngeneic tumor models, may more accurately model drug pharmacokinetics relevant to clinical exposures. However, resource constraints tend to limit their use to a few models and usually for the development of resistance to single agents. One approach to encompassing genetic diversity for a given cancer type is to use an SMT design, rather than conventional experimental designs that use 8–10 mice for control and treatment groups^[15,18]^. In this pilot study, we have evaluated SMT in the context of VAC therapy in 34 RMS models, and have used this approach to develop models with acquired resistance that can be further characterized by “omics” and biochemical methods. Essentially, the approach mimics a clinical trial in that there is no “control” arm, and the response criteria are tumor volume changes and EFS. We “enrolled” RMS models when tumors were available, and the response to one cycle of VAC was assessed in a blinded manner with respect to whether the model was established from an untreated patient or from a patient following treatment. As VAC therapy has been the backbone for the treatment of RMS for over 30 years, we assume that xenografts established from treated patients received these drugs, although it is not known if additional drugs were administered to patients before their tumors were established as xenografts.

Overall the models appear to retain drug sensitivity characteristics of patient tumors. Ten of the 13 models that had PD to VAC treatment were derived from pre-treated tumors (treated, relapse, autopsy), whereas only 2 out of 11 models established from diagnosis samples progressed on cycle 1 of VAC treatment. The models established as PDX’s from samples at diagnosis that were intrinsically resistant to VAC included two ERMS, one defined pathologically as a high-grade tumor (Rh71) and one with diffuse anaplasia (Rh85), which may relate to drug sensitivity^[30,31]^. Tumor models having EFS of 70 days or greater included 7 established from diagnosis samples, 5 listed as “treated”, and 3 models established from tumor at relapse. Clinically, patients progressing on front-line treatment may respond to phase 2 protocols, including cyclophosphamide and *vinca* alkaloids. It is also possible that prolonged passage of tumor in untreated mice could lead to loss of resistance, or overgrowth of sensitive cells. However, the data set from 34 models serves as a reference point for understanding intrinsic resistance or sensitivity and for developing acquired resistance in those models that initially respond to VAC treatment under relevant in vivo conditions.

We have used two criteria for assessing tumor sensitivity to VAC: tumor regression (partial and complete response) and EFS. Tumors having PD had EFS times of 3–8 weeks; those demonstrating PR (> 50% volume regression) had EFS that ranged from 6–14 weeks, and models having CR the EFS time ranged from 7 to > 20 weeks. Thus, as in our previous study^[17]^, there was a relatively poor correlation between the magnitude of tumor volume regression and EFS, suggesting that EFS may be a more reliable metric of tumor response in preclinical models. Clinical data also question the value of early tumor response as a prognostic indicator, as neither computed tomography and/or magnetic resonance imaging predicted outcomes for patients with rhabdomyosarcoma^[32,33]^.

We have attempted to select for acquired resistance to VAC using the SMT approach. While this pilot study is far from complete, interim analysis suggests that this approach can generate a large number of VAC-resistant models that can be analyzed for genetic/epigenetic changes associated with resistance. Thirteen xenograft models showed progressive disease in response to VAC treatment, hence were classified as intrinsically resistant. However, many of these resistant tumors had reduced EFS on subsequent cycles of therapy, thus showing increased resistance. In ongoing studies, acquired resistance has been selected within 3 to 5 cycles of VAC treatment in many tumor models initially sensitive to VAC. The approach we have taken is to define intrinsic sensitivity/resistance to VAC and to select for acquired resistance in mice. An alternative approach is to establish PDX models at diagnosis and relapse from the same patient. This is rarely possible in the context of pediatric RMS, as relapse tumor is rarely biopsied, but also engraftment into mice may select for subclones rather than represent the clonal spectrum in the patient tumor. Modeling intrinsic and acquired resistance in preclinical models has an advantage over clinical studies in that changes in gene expression can be assessed both in parental and resistant tumor, but also in response to therapy^[34]^.

Here we have defined the intrinsic sensitivity of 34 RMS models. Importantly, models intrinsically resistant to VAC, or those with acquired resistance to VAC treatment, may be particularly valuable for identifying novel agents that may be active in disease at relapse, and may have novel mechanisms of action independent of DNA damage.

## Supplementary Material

Supplemental Figure 1

## Figures and Tables

**Table 1.** Characteristics of RMS xenograft models and response to cycle 1 of VAC therapy

Model ID	Subclass	Age (years)	Gender	Site	Diagnosis or relapse	Response	EFS (weeks)
Rh10	ARMS	15	Female	Liver	Relapse	PD	6
Rh28	ARMS	17	Male	Hand	Diagnosis	MCR	> 20
Rh30R	ARMS	16	Male	Bone marrow	Relapse	CR	9
Rh41	ARMS	12	Female	Unknown	Autopsy	PD	3
Rh18	ERMS	2	Female	Perineum	Diagnosis	CR	10
Rh36	ERMS	15	Male	Paratesticular	Relapse	PR	6
Rh66	ARMS	12	Female	Axillary lymph node	Metastasis	CR	7
Rh12	ERMS	12	Male	Right buttock	Diagnosis	MCR	> 20
NCH-ERMS-1	ERMS	5	Male	orbital	No treatment	PD	5
NCH-ARMS-2	ARMS, metastatic	16	Female	Right breast	Treated	PD	4
Rh71	ERMS, high grade	17	Male	Prostate	Diagnosis	PD	6
Rh72	ERMS	3	Female	Perineal	Treated	PD	9
Rh73	ERMS	5	Male	Right infratemporal fossa mass	Treated	PD	7
Rh75	ERMS	17	Male	Pelvis recurrent (Rh71)	Treated	CR	8
Rh78	ARMS	1	Male	Right thigh	No treatment	SD	8
Rh80	ERMS anaplastic	5	Female	Stomach mass	Treated	PD	5
Rh81	ERMS	9	Male	Abdominal mass	Treated	PR	7
Rh82	ARMS	3	Male	Paratesticular	Treated	PR	10
Rh83	ERMS	4	Male	Left orbital mass	Treated	PR	14
Rh84	ARMS	2	Male	Upper lip lesion	Treated	SD	12
Rh85	ERMS diffuse anaplasia	5	Female Abdominal mass	No treatment	PD	4	
Rh86	ERMS	8	Male	Retroperitoneal mass	Treated	CR	10
Rh87	Spindle cell/sclerosing	6	Female	Oropharyngeal mass	Treated	PD	3
Rh88	ERMS	10	Male	Pelvic mass	Treated	CR	12
IRS-56	ERMS	3	Male	Buttock	Diagnosis	CR	> 20
IRS-68	ERMS	13	Male	Shoulder	Diagnosis	PR	7
SJRHB011_X	ERMS	5	Male	Infratemporal fossa	Recurrent	PD	4
SJRHB013_X	ERMS	3	Female	Perineal/bladder	Recurrent	CR	13
SJRHB010927_X1 ERMS	5	Female	Parapharyngeal	Diagnosis	PR	12	
SJRHB000026-X1	ERMS	4	Female	Pelvis	Recurrent	PD	5
SJRHB013758_X1 ERMS	4	Female	Abdomen/pelvis	Diagnosis	CR	10	
SMS-CTR*	ERMS	1	Male	Pelvis	Diagnosis	MCR	> 20
JR-1 (UK)*	ERMS	7	Female	Lung	Relapse	PD	7
RD*	ERMS	7	Female	Pelvis	Relapse	SD	11

* Indicates cell line derived xenograft. ARMS: Fusion-positive; ERMS: fusion negative; RMS: rhabdomyosarcoma; VAC: vincristine/actinomycin-D/cyclophosphamide; EFS: event-free survival; PD: progressive disease; MCR: maintained complete response; CR: complete regression; PR: partial response; SD: stable disease.
