# Supplementary material for: Approaches to identifying drug resistance mechanisms to clinically relevant treatments in childhood rhabdomyosarcoma
Source: Cancer Drug Resist. 2022 Jan 4;5(1):80–9. doi: 10.20517/cdr.2021.112 (PMC8992598; doi:10.20517/cdr.2021.112)
Supplement: Supplementary file 1 [file cdr-5-80-SupplementaryMaterials.pdf]

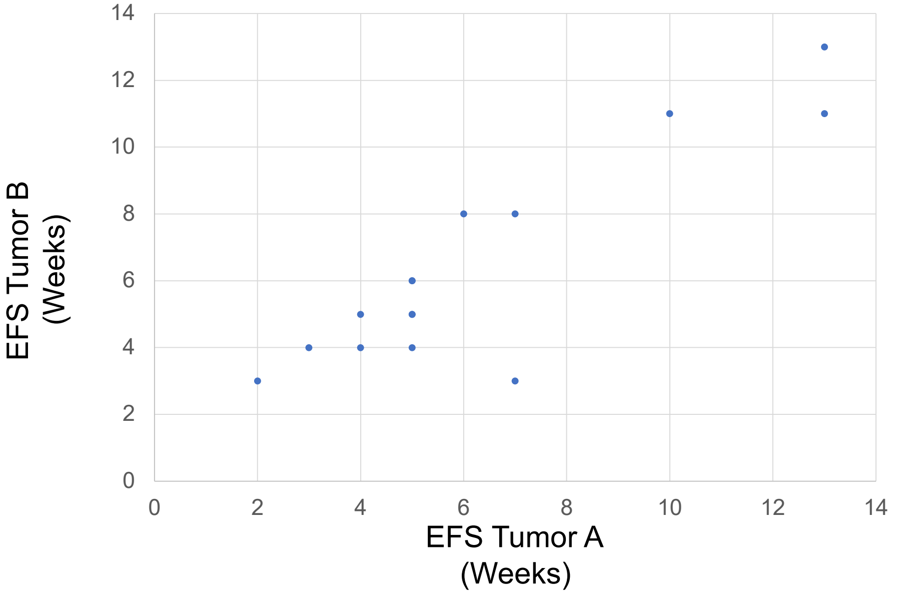


**Supplemental Figure 1.** Correlation between time to event (weeks) for the same tumor line transplanted into two mice (*n* = 16). For VAC cycle 2 of therapy, two mice were transplanted with the same line from cycle 1 in case of mortality in cycle 2. The graph shows the EFS for each tumor (designated A or B) for the same model in different mice. Overall, the EFS times are similar for both tumors for the same model. VAC: Vincristine/actinomycin-D/cyclophosphamide; EFS: event-free survival.
